# Cell membrane damage is involved in the impaired survival of bone marrow stem cells by oxidized low-density lipoprotein

**DOI:** 10.1111/jcmm.12424

**Published:** 2014-09-25

**Authors:** Xin Li, Yuan Xiao, Yuqi Cui, Tao Tan, Chandrakala A Narasimhulu, Hong Hao, Lingjuan Liu, Jia Zhang, Guanglong He, Catherine M Verfaillie, Minxiang Lei, Sampath Parthasarathy, Jianjie Ma, Hua Zhu, Zhenguo Liu

**Affiliations:** a Xiangya Hospital of Central South UniversityChangsha, Hunan, China; b Davis Heart & Lung Research Institute and Division of Cardiovascular Medicine, The Ohio State University Wexner Medical CenterColumbus, OH, USA; c Davis Heart & Lung Research Institute, Department of Surgery, The Ohio State University Wexner Medical CenterColumbus, OH, USA; d Burnett School of Biomedical Sciences, College of Medicine, University of Central FloridaOrlando, Florida, USA; e Stem Cell Institute, University of LeuvenLeuven, Belgium

**Keywords:** mesenchymal stem cells, ox-LDL, MG53, membrane damage, cell survival

## Abstract

Cell therapy with bone marrow stem cells (BMSCs) remains a viable option for tissue repair and regeneration. A major challenge for cell therapy is the limited cell survival after implantation. This study was to investigate the effect of oxidized low-density lipoprotein (ox-LDL, naturally present in human blood) on BMSC injury and the effect of MG53, a tissue repair protein, for the improvement of stem cell survival. Rat bone marrow multipotent adult progenitor cells (MAPCs) were treated with ox-LDL, which caused significant cell death as reflected by the increased LDH release to the media. Exposure of MAPCs to ox-LDL led to entry of fluorescent dye FM1-43 measured under confocal microscope, suggesting damage to the plasma membrane. Ox-LDL also generated reactive oxygen species (ROS) as measured with electron paramagnetic resonance spectroscopy. While antioxidant N-acetylcysteine completely blocked ROS production from ox-LDL, it failed to prevent ox-LDL-induced cell death. When MAPCs were treated with the recombinant human MG53 protein (rhMG53) ox-LDL induced LDH release and FM1-43 dye entry were significantly reduced. In the presence of rhMG53, the MAPCs showed enhanced cell survival and proliferation. Our data suggest that membrane damage induced by ox-LDL contributed to the impaired survival of MAPCs. rhMG53 treatment protected MAPCs against membrane damage and enhanced their survival which might represent a novel means for improving efficacy for stem cell-based therapy for treatment of diseases, especially in setting of hyperlipidemia.

## Introduction

Cell therapy with stem cells remains a viable option for tissue repair and regeneration [1–3]. Bone marrow-derived mesenchymal stem cells (BMSCs) are an attractive source for cell therapy because of the fact that BMSCs can be easily obtained without ethical concerns, and conveniently expanded *ex vivo* to clinical scales in a short time with minimal loss of potency and little (if any) inherent immunogenicity for adverse immune reactions because of their immunosuppressive and/or immunomodulatory properties [4–6]. However, cell therapy with BMSCs has faced serious challenges because of low viability of the implanted cells [7–10]. Recent studies have shown that less than 1% of systemically administered BMSCs are still present for longer than a week in various organs including lung, heart, kidney, liver, spleen, and gut following injection [6,11–14]. Although some factors have been considered to cause the poor survival of transplanted cells, such as inflammation and hypoxia, the exact mechanism(s) remains largely unknown.

Oxidized-low-density lipoprotein (ox-LDL) is a natural product in human blood. The serum ox-LDL concentration was estimated to be 0.7 mg/dl in healthy individuals. The serum ox-LDL level was 1.72 mg/dl and 2.36 mg/dl for the patients with stable coronary artery disease (CAD) and the ones with acute coronary syndrome, respectively [15–17]. Ox-LDL exhibits significant effects on progenitor cell especially endothelial progenitor cell (EPC) including apoptosis induction and suppression of function and therapeutic potential [18–26]. A variety of mechanisms are involved in the actions of ox-LDL on the progenitor cells, including up-regulation and/or activation of MAPK and LOX-1 (a membrane ox-LDL receptor) pathways. In addition, the cytokines produced by macrophage, leucocyte and platelet could indirectly modify the function of stem cells in the presence of ox-LDL [27].

Previously, we showed that ox-LDL inhibited the proliferation and differentiation of BMSCs and induced their apoptosis *in vitro* [28,29]. We also observed that a significant amount of reactive oxygen species (ROS) was generated by ox-LDL *in vitro*, indicating ROS might be the cause of cell death. To test this hypothesis, we co-treated cells with N-acetylcysteine (NAC), a ROS scavenger, to completely block ROS production. Interestingly, while NAC could protect BMSCs when ox-LDL was low (5 μg/ml or less), the protective effects of NAC was abolished when ox-LDL was increased to 10 μg/ml or higher. Of note, low level of ox-LDL predominantly inhibited proliferation and differentiation of BMSCs, while it largely caused cell death at a level of 20 μg/ml (comparable to serum ox-LDL level for CAD patients). These data indicated that a ROS-independent mechanism(s) might contribute to ox-LDL-induced BMSCs death.

Plasma membrane damage repair is a critical process for cell survival. Our previous studies have established that MG53 is an essential gene for membrane repair [30]. MG53, also known as TRIM72, is a muscle-specific member of tripartite motif-containing (TRIM) superfamily which comprises a RING finger domain, one or two B-boxes and one or two coiled-coil domain [31]. MG53 acts as a sensor of oxidation to oligomerize and then recruit intracellular vesicles to the injury site to allow for membrane patch formation [30]. In addition, we also showed that recombinant human MG53 (rhMG53) could ameliorate pathology of muscular dystrophy mice when it was administrated through intravenous injection [32], suggesting a potential application of rhMG53 in the treatment of diseases with compromised membrane repair, such as cardiac diseases and muscular dystrophy.

In this study, we suggested that plasma membrane damage might be a novel mechanism for ox-LDL-induced cell death. We first demonstrated that ox-LDL could indeed induce membrane injury to BMSCs by quantitatively detecting the release of intracellular lactate dehydrogenase (LDH) from the cell and the entry of fluorescent dye FM1-43 into the cell. To protect BMSCs against ox-LDL-induced membrane injury, rhMG53 was used to treat the cells. We showed that rhMG53 could significantly protect membrane injury to BMSCs by ox-LDL, and enhance their survival. Thus, we demonstrated for first time that membrane damage contributed to ox-LDL-induced death of BMSCs and treatment with rhMG53 might represent a novel approach for enhancing BMSC survival after transplantation.

## Materials and Methods

### Preparation of LDL and ox-LDL

Native LDL was prepared from the plasma in healthy human participants by sodium bromide stepwise density gradient centrifugation. Ox-LDL was prepared by the exposure of native LDL to copper sulphate (5 μM) at 37°C for 3 hrs. The reaction was stopped by adding EDTA (0.25 mM, final concentration) as described [29,33,34]. Thiobarbituric acid reactive substances (TBARS) were used as an index of the degree of LDL oxidation. The TBARS value for ox-LDL was between 40 and 50 nmol malondialdehyde/mg protein. TBARS were not detectable in native LDL.

### Preparation of rhMG53

*Escherichia coli* fermentation was used to obtain high quality (>97% purity) rhMG53 protein as described [32]. The membrane protective activity of rhMG53 (EC50) from each preparation was determined with established micro-glass bead injury assay as described [32,35]. The rhMG53 concentration for this study was its EC50 as determined by micro-glass bead injury assay.

### Cell culture

Rat bone marrow multipotent adult progenitor cells (MAPCs) were prepared and characterized in Dr. Verfaillie's laboratory in the Stem Cell Institute at the University of Leuven, Leuven, Belgium. Phenotypically, these cells were positive for Oct-4, Rex-1, c-Kit, and Pdgfr-a, and negative for Sca-1, CD34, CD45, Sox-2 and Nanog [29,36,37]. The cells were cultured with ox-LDL (from 0 to 20 μg/ml) for up to 48 hrs with and without rhMG53 (50–80 μg/ml depending on the EC_50_ of each protein preparation) to determine the cell growth and survival. Native LDL and PBS served as the controls. To determine the involvement of ROS in the actions of ox-LDL, experiments were repeated when NAC (1 mM) was present.

### Cell proliferation assay

Rat MAPCs were seeded in a 96-well plate at a density of 1000 cells/well in the presence of ox-LDL (10 μg/ml) for 12, 24, 36 and 48 hrs. When 20 μg/ml ox-LDL was used, the cells were cultured for 24 hrs at a density of 3000 cells /well since the cells could die out quickly. Bovine serum albumin (BSA) was used as control. To evaluate the effect of NAC (1 mM) or rhMG53 (50–80 μg/ml depending on the EC_50_ of each protein preparation) on cell proliferation and survival, each reagent was mixed in the culture medium 5 min. before exposure to ox-LDL. At each time-point, the cells were prepared for proliferation assay using BrdU proliferation Assay Kit (Calbiochem, San Diego, CA, USA) as per manufacturer's instruction. All samples were prepared in duplicates.

### Measurement of ROS formation

Production of extracellular ROS from ox-LDL in the culture system was quantitatively determined using electron paramagnetic resonance (EPR) spectroscopy as described [38], and intracellular ROS was detected using ROS detection reagent 2′,7′-dichlorodihydrofluorescein diacetate (H2DCFDA; Life Technologies D399, Carlsbad, CA, USA) dissolved in ethanol (0.5 mg/100 μl) as per the manufacturer's instruction. Medium with PBS was used as background with native LDL as control. To evaluate the effect of NAC on ROS production from ox-LDL, NAC (1 mM) was mixed with the medium 5 min. prior to adding ox-LDL.

### Micro-glass beads cell membrane damage assay

The assay for cell membrane damages was modified from our established assay for measuring rhMG53 activity in HEK293 cells [32,35]. In brief, rat MAPCs (1 × 10^6^ cells/ml) were treated with extracellular rhMG53 or control protein (BSA), and then exposed to mechanical plasma membrane damage by orbital shaking with micro-glass beads. The level of membrane damage was measured by LDH release from the cells using a LDH Detection Kit (Takara Mountain View, CA, USA) at 490 nm as per manufacturer's instruction.

### Quantification of LDH released from the cells induced by ox-LDL

Rat MAPCs were seeded in a 24-well plate at a density of 1 × 10^4^ cells/cm^2^ in the presence of ox-LDL (from 0 to 20 μg/ml) for up to 48 hrs with or without rhMG53. PBS, native LDL, and BSA were used as controls. The amount of LDH released from the cells was measured with LDH Cytotoxicity Detection Kit (cat. #MK401; Takara) according to the manufacturer's instruction. Rat MAPCs were seeded in a 24-well plate at a density of 1 × 104 cells/cm^2^ in the presence of ox-LDL (from 0 to 20 μg/ml) for 6, 12 and 24 hrs. PBS and native LDL were used as controls. After incubating the cells overnight to allow the cells to adhere, the medium was replaced with fresh culture medium containing different concentrations of ox-LDL. The medium of each well was collected at each time-point, and centrifuged at 1000 g for 5 min. Then, the supernatant was carefully transferred into corresponding wells of a clear 96-well flat bottom microtiter plate (100 μl/well), and mixed with detection reagent (1:1) for 30 min. at room temperature. The absorbance of the samples was measured using an ELISA reader at 490 nm with the reference wavelength of 690 nm. To determine if rhMG53 could protect the cell membrane against ox-LDL, the experiments were repeated with the cells with the presence of rhMG53. BSA was used as the control protein.

### FM1-43 dye entry detection

Rat MAPCs were seeded in the 35 mm Glass Bottom Dishes at a density of 1 × 10^4^ cells/cm^2^ and incubated over overnight. The cells were then treated with ox-LDL 10 μg/ml with or without rhMG53 (at EC_50_) for 24 hrs with PBS and BSA as controls. After rinsing with Tyrode's solution, the cells were mixed with FM1-43 dye. FM1-43 is membrane impermeable and becomes fluorescent when it enters injured cells and binds to cellular lipids [30,32,35,39,40]. Dye entry into the cells was monitored continuously with fluorescence confocal microscope (Zeiss LSM780 Oberkochen, Germany) immediately after mixing with the dye. Consecutive live cell images were obtained at an interval of 4.1 sec./frame for 100 frames.

### Statistical analysis

These data were expressed as mean ± SE in all experiments and statistically analyzed with unpaired Student's *t*-test (two-sided) for two group comparison or one-way anova (SigmaStat 2.03; Aspire Software International, Ashburn, VA, USA) followed by *post hoc* conservative Tukey's test for multiple comparisons of three or more groups to minimize Type I error. When a two-tailed *P* < 0.05, the difference was considered statistically significant.

## Results

### Ox-LDL significantly decreased the growth and survival of MAPCs

**Fig. 1:**
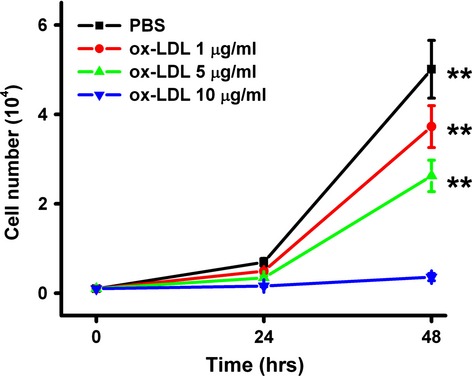
ox-LDL impairs MAPCs growth. When MAPCs were cultured in the presence of ox-LDL (0–10 μg/ml for 0–48 hrs), the cell number was dramatically decreased in a concentration- and time-dependent manner. When the cells were exposed to 20 μg/ml ox-LDL, almost all the cells died within 24 hrs of culture (data not shown). **P < 0.01 as compared with control (*n* = 3 independent experiments, data presented as means ± SEM).

Exposure to ox-LDL dramatically decreased the number of MAPCs in a concentration-dependent manner (Fig. 1). When ox-LDL concentration increased to 10 μg/ml, there was no increase in the cell number in the culture system. When increased to 20 μg/ml, almost all the cells died within 24 hrs, suggesting that ox-LDL at concentration compatible to that in CAD patients significantly impaired the survival of MAPCs. Native LDL had no effect on cell population in culture.

### NAC completely blocked ROS production from ox-LDL, but only partially protected MAPCs against ox-LDL

**Fig. 2:**
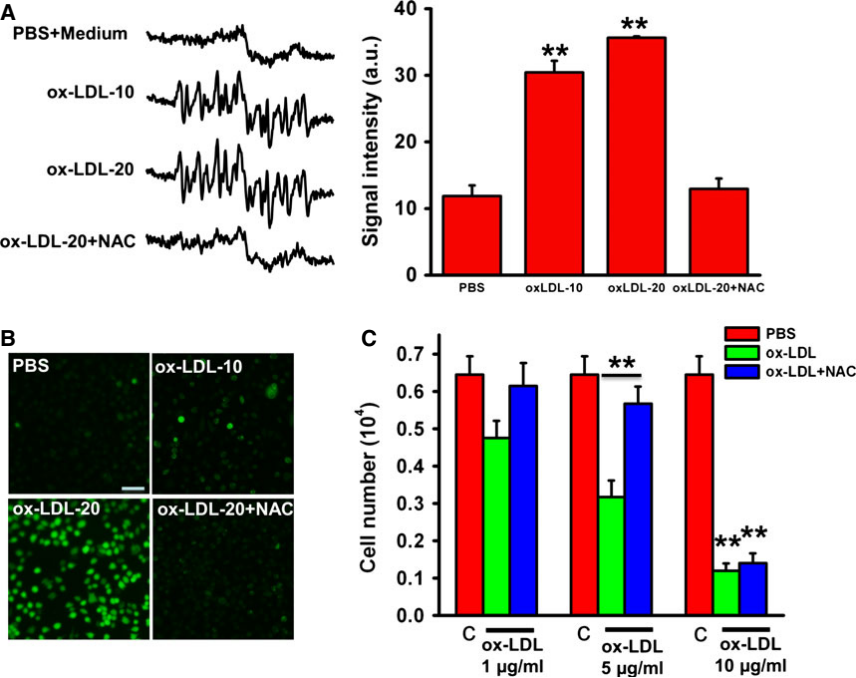
NAC blocks ROS generation by ox-LDL, but fails to rescue cell death at high concentration of ox-LDL. Ox-LDL (10 or 20 μg/ml) increased ROS production in the culture media dose dependently as detected by EPR. The antioxidant NAC (1 mM) completely blocked ROS production from ox-LDL (**A**). Intracellular ROS formation detected with 2′,7′-dichlorodihydrofluorescein dictate (H2DCFDA) was also significantly increased in MAPCs exposed to ox-LDL in a dose-dependent manner that was also effectively prevented with NAC (scale bar: 80 μm; **B**). However, NAC treatment only prevented the reduction in cell number of MAPCs by ox-LDL at a concentration of 5 μg/ml or less, but failed to protect the cells against ox-LDL at the concentration of 10 or higher (C). **P < 0.01 as compared with control (*n* = 3 independent experiments, data presented as means ± SEM).

A significant amount of extracellular (by EPR) and intracellular ROS (by H2DCFDA assay) was generated from ox-LDL. NAC treatment completely blocked both extracellular and intracellular ROS production from ox-LDL (up to 20 μg/ml, Fig. 2A and B). Interestingly, NAC effectively prevented the inhibitory effect of ox-LDL at 1 or 5 μg/ml on cell population in culture. However, NAC had no protective effect on MAPCs against ox-LDL at 10 μg/ml or higher (Fig. 2C). Thus the effect of ox-LDL on MPACs was mediated through ROS production at low concentrations, while independent of ROS formation at higher but clinically relevant concentrations *in vitro*. In addition, to determine that the deleterious effects are ox-LDL specific, we treated MAPCs with both native LDL (non-oxidized form of LDL) and saturated LDL (chemically modified oxidized resistant form of LDL). We observed that both of them failed to introduce cell death and ROS formation (data not shown).

### Ox-LDL induced cell membrane damage to MAPCs

**Fig. 3:**
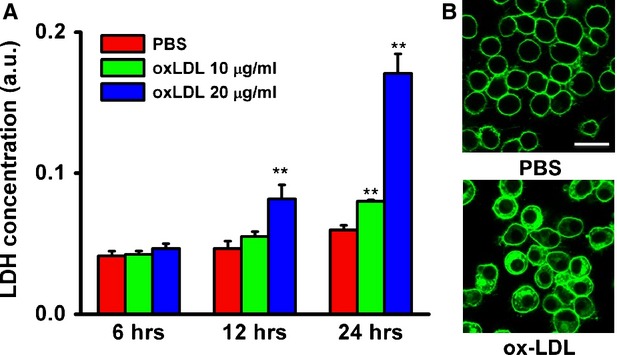
ox-LDL induces plasma membrane injury to MAPCs. After 24 hrs of culture with ox-LDL (10 and 20 μg/ml), there was a significant increase in the release of intracellular lactate dehydrogenase (LDH) from MAPC in the conditioned media in a time- and dose-dependent manner (**A**) as compared with PBS the control. Exposure to ox-LDL (10 μg/ml) also substantially increased the entry of fluorescent dye FM1-43 into the cells as demonstrated with the live confocal microscope. As expected, no dye entered into the control cells with intact membrane (**B**). **P < 0.01 as compared with control at the same time-point (*n* = 3 independent experiments, data presented as means ± SEM; scale bar: 20 μm).

Release of intracellular LDH from cell and FM1-43 dye entry into cell are well-established markers for membrane injury. Exposure to ox-LDL led to a significantly increased LDH release from MAPCs in conditioned media in a time- and dose-dependent manner (Fig. 3A), suggesting that ox-LDL indeed induced membrane damage to MAPCs. Membrane damage by ox-LDL was further confirmed by entry of fluorescent dye FM1-43 into the cells that was quantitatively and dynamically analyzed with a live cell imaging assay. As expected, no dye entered into the control cells with intact membrane. However, a significant and rapid dye accumulation inside the cells was observed when exposed to ox-LDL (10 μg/ml, Fig. 3B).

### MG53, not NAC, reduced cell membrane damage to MAPCs by ox-LDL

**Fig. 4:**
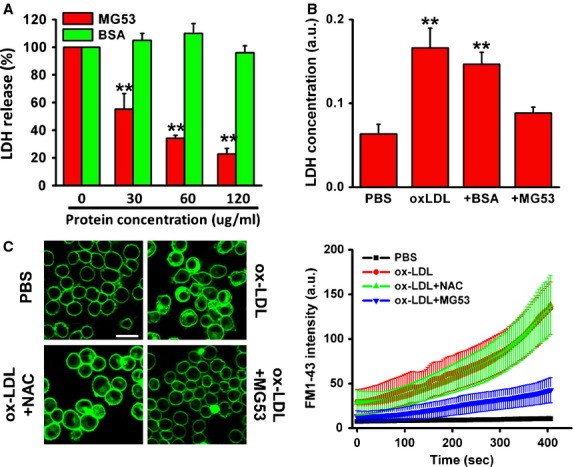
rhMG53 protects MAPCs against membrane damage following both mechanical injury and ox-LDL treatment. When MAPCs were exposed to mechanical plasma membrane damage with glass beads, a significant amount of intracellular lactate dehydrogenase (LDH) was released from the cells. LDH release was significantly decreased from MAPCs exposed to the mechanical membrane injury when rhMG53 was present in a dose-dependent manner as compared with BSA control (**A**). The EC50 of rhMG53 was determined to be the protein concentration that reduced the total LDH release by 50%. Treatment of MAPCs with rhMG53 at EC50 concentration significantly decreased LDH release induced by ox-LDL (20 μg/ml) from the cells, while there was no effect of BSA on ox-LDL-induced LDH release (**B**). Quantitative and dynamic analysis with a quantitative live cell imaging assay showed that exposure to ox-LDL (10 μg/ml) dramatically increased FM1-43 dye accumulation inside the cells that was significantly reduced by rhMG53, but not by BSA or NAC. The dynamic of FM1-43 dye entry was analyzed by imageJ (more than 200 cells were analyzed for each condition and each experiment) (**C**). **P < 0.01 as compared with rhMG53 treatment (*n* = 3 independent experiments, data presented as means ± SEM; scale bar: 20 μm).

MG53 is a specific membrane repairing protein [30,35,40–45]. To ensure that MG53 could also repair membrane damage to MAPCs, the cells were exposed to mechanical injury with micro-glass beads with and without rhMG53. As expected, rhMG53 effectively protected the cells against mechanical membrane damage as reflected by a significant reduction in LDH release in a dose-dependent manner (Fig. 4A). rhMG53 also significantly decreased LDH release from MAPCs exposed to ox-LDL along with a substantial reduction in FM1-43 dye entry into the cells (Fig. 4B and C, Movies S1–S4). However, NAC treatment did not prevent dye entry into the cells exposed to ox-LDL (Fig. 4C), thus no protection against ox-LDL-induced membrane damage.

### MG53 partially reversed ox-LDL-induced inhibition of cell proliferation and cell death

**Fig. 5:**
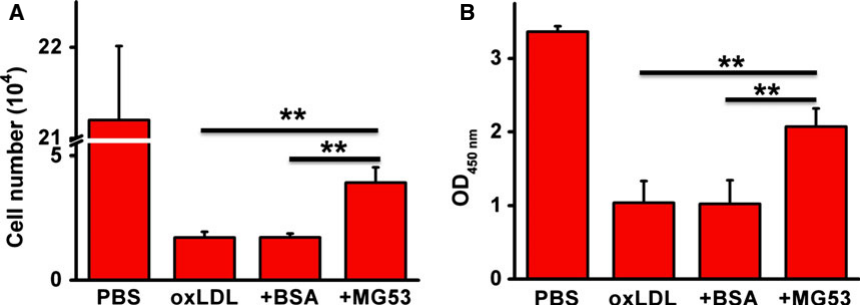
rhMG53 rescues cell death induced by ox-LDL. When MAPCs were cultured with ox-LDL at the concentration of 20 μg/ml, almost all the cells died within 24 hrs. However, when rhMG53 was added into the culture system, the cell population was significantly increased to 20% of the control after 24 hrs of culture with 20 μg/ml ox-LDL. BSA at the same concentration as rhMG53 had no effect on cell population (**A**). BrdU assay showed that ox-LDL significantly inhibited the proliferation of MAPCs that was substantially reversed by rhMG53 (**B**; ox-LDL at 20 μg/ml with 3000 cells/well with 24 hrs of culture). **P < 0.01 as compared with rhMG53 treatment (*n* = 3 independent experiments, data presented as means ± SEM).

After 24 and 48 hrs of culture with ox-LDL (10 μg/ml), the number of MAPCs in the system remained unchanged (500/cm^2^), while the cells grew rapidly in the control group with PBS (Fig. 1). When ox-LDL concentration increased to 20 μg/ml, the majority of the cells were dead after 24 hrs. In the presence of rhMG53, the cell population was significantly increased to 50% and 20% of the control after 24 hrs of culture with 10 and 20 μg/ml ox-LDL, respectively (Fig. 5A). BrdU assay demonstrated that ox-LDL significantly inhibited the proliferation of MAPCs that was substantially reversed by rhMG53 (Fig. 5B).

## Discussion

The effects of ox-LDL on its target cells are variable and complex [46–52]. Low ox-LDL level stimulates proliferation, migration and adhesion of mouse bone marrow mesenchymal stem cells [53,54]. It promotes proliferation of macrophage and smooth muscle cell, and inhibits apoptosis of macrophage and monocyte [50,55–60]. On the other hand, high level of ox-LDL inhibits proliferation and promotes apoptosis of endothelial cell and EPCs [23,61,62] (for the sake of discussion, ox-LDL concentrations above the serum level of healthy individuals without CAD or hyperlipidemia were considered ‘high’ ox-LDL levels). Recently, we demonstrated that ox-LDL inhibited proliferation, induced apoptosis of BMSCs, and attenuated their endothelial differentiation [28,29]. In this study, we demonstrated that ox-LDL produced significant damage to cell membrane of MAPCs and impaired their survival *in vitro* as demonstrated by increased LDH release and FM1-43 dye entry into the cells. The membrane repair protein rhMG53 significantly protected MAPCs against ox-LDL-induced membrane injury, and significantly improved their survival. To our knowledge, this was the first time to show that membrane damage played an important role in the impaired survival of BMSCs by ox-LDL.

Ox-LDL interacts with a variety of cells *via* multiple mechanisms including activating mitogen-activated protein kinase and E-cadherin/β-catenin/Tcf pathway, promoting LOX-1, and MCP-1 expression, downregulating E-selectin and integrin a(v)b(5) expression, and inhibiting PI3K/Akt signalling [21,28,53,54,63,64]. Another important mechanism for the actions of ox-LDL is ROS formation and oxidative stress. ROS generation in the peripheral blood monocytes is increased in hyperlipidemic patients with elevated plasma ox-LDL level [65]. ROS formation from ox-LDL was involved in its actions on MAPCs, including specific stem cell marker Oct-4 expression, proliferation of MAPCs, and their endothelial differentiation [28,29]. In this study, we observed that ox-LDL, enhanced intracellular and extracellular ROS production with significantly reduced number of MAPCs *in vitro*. NAC completely blocked ROS formation from ox-LDL, but only prevented the effect of low dose ox-LDL on the growth of MAPCs without protection against ox-LDL-induced death of MAPCs at a concentration of 10 μg/ml or higher. These data suggested that ox-LDL-induced inhibition of cell proliferation of MAPCs was dependent on ROS formation, while ox-LDL-induced cell death of MAPCs at the concentration comparable to serum ox-LDL levels in CAD patients was independent of ROS production.

Membrane integrity is critical to cell survival and function including growth, differentiation and mobility [66–69]. Lipids and phospholipids are important part of cell membrane structure. Ox-LDL interacts with cell membrane lipids and modifies cell function. Exposure of endothelial cell to ox-LDL increases membrane stiffness with changes in their contractility and angiogenic potential because of disruption of lipid packing of cholesterol-rich membrane domains [70–72]. In this study, we showed for the first time that ox-LDL produced significant membrane damage to MAPCs that contributed to their death *in vitro*. The role of membrane damage in mediating ox-LDL-induced cell death was further confirmed by the finding that the specific membrane repair protein rhMG53 significantly reduced cell membrane damage by ox-LDL, improved the survival of MAPCs and their proliferation in the presence of ox-LDL.

Repair of acute disruptions to the plasma membrane is an important aspect of normal cell physiology. MG53 is a critical acute membrane repair molecule. Our previous study has shown that purified rhMG53 could effectively repair cell membrane damages to skeletal muscles both *in vitro* and *in vivo* [32]. In this study, we showed that rhMG53 also effectively protect MAPCs against mechanical membrane damage by microglasses and reduced the membrane damages to MAPCs by ox-LDL. Of note, rhMG53 only partially reversed the effect of ox-LDL on cell death of MAPCs, suggesting there is still room for enhancing efficacy for MG53-mediated cell protection. For example, Kohr *et al*. elegantly demonstrated that oxidative stress induced oxidation of MG53 at cysteine 144 residue greatly attenuated its stability [44]. Thus, we could generate mutant rhMG53 (*e.g*. MG53_C144S_) to enhance the half-life of MG53 in oxidative environment (ox-LDL), or to modify MG53 with s-nitrosylation [44]. Clearly, other mechanism(s) is also involved in the impaired survival of MAPCs by ox-LDL. Indeed, we observed that phosphorylated Akt level was significantly decreased in MAPCs exposed to ox-LDL, suggesting that Akt signalling was also important in the survival of MAPCs (data not shown).

The finding that membrane damage contributed to impaired survival of MAPCs by ox-LDL might have significant clinical relevance. One major challenge for cell therapy with BMSCs is the poor *in vivo* survival. Identification of the factors contributing to *in vivo* cell loss and related mechanism(s) could be a critical initial step to enhancing cell survival and therapeutic efficacy. Data from this study could provide important information on patient selection and preparation for cell therapy with BMSCs. The majority of patients with myocardial infarction or stroke who could be ideal candidates for cell therapy have CAD and hyperlipidemia with elevated ox-LDL level. Optimal control of hyperlipidemia could improve the survival of transplanted BMSCs and enhance the outcome for hyperlipidemic patients. Alternatively, a membrane protection agent like rhMG53 could serve as an effective strategy to minimizing the loss of transplanted BMSCs, especially for hyperlipidemic patients. Investigations are needed to evaluate the role of ox-LDL and hyperlipidemia in the survival of transplanted BMSCs and related mechanism(s) *in vivo*.

Of course, our present study cannot address every question regarding MG53-mediated stem cell protection. For example, in addition to membrane damage repair, are there other mechanisms involved in beneficial effects of MG53? Can MG53 protect ox-LDL-induced stem cell injury *in vivo*? For the first question, we have had preliminary data suggesting that the Akt prosurvival pathway might be activated by exogenous MG53 application. However, further studies are required to determine how extracellular application of MG53 can activate intracellular signal. Furthermore, we will take advantage of hyperlipidemia animal models developed in our group to test the *in vivo* function of MG53 on stem cell protection.

In conclusion, we demonstrated that ox-LDL significantly decreased the survival of MAPCs in part due to induction of membrane damage *in vitro*. The specific membrane repair protein rhMG53 protected the cells against ox-LDL-induced membrane damages and enhanced their survival. Data might have important clinical implication for cell therapy with BMSCs especially for hyperlipidemic patients.

## Supporting information

**Movie S1** MAPCs treated with PBS as control.

**Movie S2** MAPCs treated with ox-LDL (10 μg/ml).

**Movie S3** MAPCs treated with ox-LDL (10 μg/ml) + NAC (1 mM).

**Movie S4** MAPCs treated with ox-LDL (10 μg/ml) + rhMG53 (EC50).
