# Supplementary material for: Cell membrane damage is involved in the impaired survival of bone marrow stem cells by oxidized low-density lipoprotein
Source: J Cell Mol Med. 2014 Sep 25;18(12):2445–53. doi: 10.1111/jcmm.12424 (PMC4302650; doi:10.1111/jcmm.12424)
Supplement: Supplementary file 1 — Movie S1 MAPCs treated with PBS as control. [file jcmm0018-2445-sd1.docx]

Supplemental movies

Dye entry into the cells was monitored continuously with fluorescence confocal microscope (Zeiss LSM780) immediately after mixing with the dye. Consecutive live cell images were obtained at an interval of 4.1 s/frame for 100 frames. Movie s1: MAPCs treated with PBS as control. Movie s2: MAPCs treated with ox-LDL (10 μg/ml). Movie s3: MAPCs treated with ox-LDL (10 μg/ml) + NAC (1 mM). Movie s4: MAPCs treated with ox-LDL (10 μg/ml) + rhMG53 (EC50).
